# Intermittent radiotherapy as alternative treatment for recurrent high grade glioma: a modeling study based on longitudinal tumor measurements

**DOI:** 10.1038/s41598-021-99507-2

**Published:** 2021-10-12

**Authors:** Sarah C. Brüningk, Jeffrey Peacock, Christopher J. Whelan, Renee Brady-Nicholls, Hsiang-Hsuan M. Yu, Solmaz Sahebjam, Heiko Enderling

**Affiliations:** 1 Department of Biosystems Science and Engineering, ETH Zürich, Basel, Switzerland; 2 Swiss Institute for Bioinformatics (SIB), Lausanne, Switzerland; 3 Department of Radiation Oncology, H. Lee Moffitt Cancer Center, Tampa, FL USA; 4 Department of Cancer Physiology, H. Lee Moffitt Cancer Center, Tampa, FL USA; 5 Department of Integrated Mathematical Oncology, H. Lee Moffitt Cancer Center, Tampa, FL USA; 6 Department of Neuro-Oncology, H. Lee Moffitt Cancer Center, Tampa, FL USA; 7 Department of Oncologic Sciences, University of South Florida, Tampa, FL USA

**Keywords:** Cancer models, Applied mathematics, Computational science

## Abstract

Recurrent high grade glioma patients face a poor prognosis for which no curative treatment option currently exists. In contrast to prescribing high dose hypofractionated stereotactic radiotherapy (HFSRT, $\ge 6$ Gy $\times$ 5 in daily fractions) with debulking intent, we suggest a personalized treatment strategy to improve tumor control by delivering high dose intermittent radiation treatment (iRT, $\ge 6$ Gy $\times$ 1 every 6 weeks). We performed a simulation analysis to compare HFSRT, iRT and iRT plus boost ($\ge 6$ Gy $\times$ 3 in daily fractions at time of progression) based on a mathematical model of tumor growth, radiation response and patient-specific evolution of resistance to additional treatments (pembrolizumab and bevacizumab). Model parameters were fitted from tumor growth curves of 16 patients enrolled in the phase 1 NCT02313272 trial that combined HFSRT with bevacizumab and pembrolizumab. Then, iRT +/− boost treatments were simulated and compared to HFSRT based on time to tumor regrowth. The modeling results demonstrated that iRT + boost(− boost) treatment was equal or superior to HFSRT in 15(11) out of 16 cases and that patients that remained responsive to pembrolizumab and bevacizumab would benefit most from iRT. Time to progression could be prolonged through the application of additional, intermittently delivered fractions. iRT hence provides a promising treatment option for recurrent high grade glioma patients for prospective clinical evaluation.

## Introduction

Patients with recurrent high-grade glioma (rHGG), such as glioblastoma, face a dismal prognosis with median overall survival rates of less than 1 year^1,2^. This is likely related to the biological nature of these types of tumors which are characterized as fast growing, infiltrating, and frequently multifocal disease^3^. The diffuse nature of these tumors implies that any localized treatment, such as surgery or radiotherapy, inevitably fails to treat all (microscopic) disease and recurrences may hence occur either at the primary, or a distal location within the brain. According to National Comprehensive Cancer Network (NCCN) guidelines^4^ there is no well defined standard of care for these patients and treatment options are limited. Hence, treatment strategy is often suggested on an individualized basis. These include re-resection of the tumor, systemic therapy such as bevacizumab, lomustine, or temozolomide, and palliative re-irradiation. Notably, re-irradiation in the rHGG setting may be considered as a Category 2B option implying that there is NCCN consensus that this intervention is appropriate based upon lower level evidence^4^. Recently, alternative approaches incorporating immunotherapy^5^ have been tested for rHGG in several clinical trials (see Laub et al. for an extensive review^6,7^) but the efficacy of this treatment could not be demonstrated. Inevitably, rHGG tumors develop resistance to these systemic therapies.

A number of modeling approaches have investigated the responses of (low grade) glioma and oligodendroglioma in clinical^8–12^ and preclinical^13^ settings. Pérez-García et al. specifically investigated the implications of changing the radiotherapy treatment fractionation to highly protracted delivery for treatment of low grade gliomas^11^ and oligodendrogliomas^9^. These analyses provided very promising theoretical results predicting a potential survival benefit in case of intermittent radiotherapy delivery. Intermittent RT is suggested to delay the emergence of treatment-resistant and more aggressive clones. Moreover, intermittent delivery may widen the therapeutic window between normal tissue complication and tumor control due to a superior repair capacity of normal tissue. This could enable dose escalation without necessarily increasing toxicity. There also exists increasing evidence for synergistic combination of radiotherapy with immune checkpoint inhibitors^14^. Although the underlying mechanisms of action remain subject to investigation, the immunogenic effect of radiation has been demonstrated in numerous (pre) clinical studies^15^. It is suggested that radiation-induced damage triggers innate immune receptors leading to enhanced antigen presentation and immune cell activation^16^. As such, intermittently delivered radiotherapy could provide repeated immune system stimuli and could potentially be further enhanced by combination with immune checkpoint inhibitors^14^. In summary, there exists a strong motivation to further investigate protracted fractionation in rHGG.

In our recent phase 1 clinical trial (NCT02313272, 05/12/2014) rHGG patients were treated with a combination of hypofractionated stereotactic radiotherapy (HFSRT; $\ge 6$ Gy $\times$ 5 fractions), bevacizumab [antibody against vascular endothelial growth factor (VEGF)] and pembrolizumab (anti PD1 antibody)^17^. This study demonstrated safety in terms of adversarial side effects for this particular protocol. Although efficacy was not the primary endpoint, the response results were promising; yet median time to progression remained below 1 year^17^. In this trial, HFSRT was given in doses with maximum log cell kill intent over 1 week (consistent with current practice and trials for rHGG re-irradiation studies^18^). While this dose fractionation strategy can be effective in eradicating cancer cells, it may select for radiation-resistant subclones by preferential killing of radiosensitive subclones through an ecological-evolutionary process called competitive release^19–21^. The current protocol of maximum tolerable dose did not account for such evolutionary dynamics, and every patient inevitably developed resistance. Furthermore, HFSRT given upfront prevented the possibility to re-irradiate any additional (local or distant) recurrences, and provided only a single immune stimulus for anti PD1 treatment. Glazar et al. developed a mathematical model describing the dynamic growth response of rHGG to a combination of bevacizumab and pembrolizumab aiming to predict the onset of treatment resistance^12^. Yet their approach did not account for the effects of radiotherapy and how adaptation to the fractionation schedule may improve patient outcome.

Here, we continue Glazar et al.’s preliminary work and investigate through mathematical modeling an alternative, intermittent approach for radiotherapy treatment schedules. This approach is motivated by the assumption that, for this group of patients, tumor management would prolong time to progression compared to (failed) tumor eradication. The rationale of evolutionary principles-guided intermittent treatment approach is to maintain a treatment-sensitive population that competes for resources with resistant cells and thus slows the expansion of a resistant clone, thereby prolonging time to progression^22,23^. Moreover, when delivering radiotherapy using an intermittent schedule, treatment dosing and irradiation volume can be adapted based on observed responses.

In this manuscript we describe a mathematical model, using only three patient-specific parameters, that is suitable to fit clinically observed longitudinal, volumetric tumor growth in patients enrolled in the NCT02313272 trial. We use this model to simulate alternative, intermittent treatment schedules to determine whether these provide superior and personalizable alternatives to the current HFSRT for protocol for rHGG.

## Materials and methods

### Patient cohort

Patients with rHGG included in this modeling study (n = 16) were treated at the Moffitt Cancer Center, FL between August 2015 and March 2018 as part of a phase I clinical trial (NCT02313272, 05/12/2014)^17^. All patients provided written consent and the treatment protocol was approved by the institutional review board (IRB study #: Pro00014674 and # 00000971). Following optional surgical resection, all patients received HFSRT (protocol of 6 Gy $\times$ 5 delivered as five consecutive, daily fractions). Here, treatment was prescribed as 30–35 Gy to the planning target volume (PTV) with a simultaneously integrated boost to the gross tumor volume (GTV) of $D_{95\%} = 30-40$ Gy. All treatment plans were calculated in iPlan (Version 1.1, Brainlab, Munich, Germany) and were delivered as intensity modulated radiotherapy treatments using volumetric modulated arc therapy with image guidance. Planned doses were summarized in terms of generalized equivalent uniform dose (gEUD)^24,25^ and near minimum dose $D_{98\%}$, delivered to the PTV. gEUD ranged between 31.3 and 37.5 Gy, whereas the corresponding near minimum dose $D_{98\%}$ was between 28.8 and 35.9 Gy (see Supplementary Table S1 for specific total dose per patient). gEUD calculations were performed in MATLAB (version 2020a) using an exponent (Lyman parameter) of − 10 as suggested previously^26^. The gEUD accounts for dose inhomogeneity, whereas the PTV captures geometric delivery uncertainties across the gross tumor volume, providing the basis for volumetric response evaluation.

**Figure 1:**
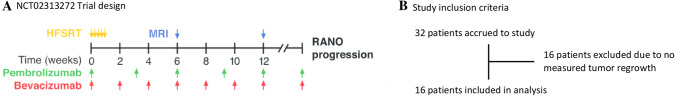
Overview of the used data. (**A**) Schematic of the NCT02313272 protocol indicating the imaging and treatment time point for this triple combination therapy trial. (**B**) Out of the 32 trial participants only those 16 with monitored tumor progression were included in this analysis.

In addition to HFSRT, all patients received the VEGF inhibitor bevacizumab (10 mg/kg, intravenously delivered every 2 weeks) and the anti PD1 antibody pembrolizumab (100 mg or 200 mg, intravenously applied every 3 weeks until progression; Fig. 1A). Tumor volume was assessed pre-treatment and approximately every 6 weeks (median 42 days, standard deviation 38 days) using T1-weighted, contrast enhanced magnetic resonance imaging (3T MRI, 1.5 mm slice thickness). The region of hyperintensity on post-contrast T1-weighted MR images (T1 post) was contoured by a neuro-radiation oncologist as the GTV. Where required, additional MRI sequences such as T2-weighted and/or FLAIR imaging were used to accurately assess this GTV, especially when there was significant tumor associated edema. A 5 mm expansion was made from the GTV to create the PTV.

Response was assessed every 6 weeks per Response Assessment in Neuro-Oncology (RANO^27^) criteria. Radiographic progression is defined as 25$\%$ or greater increase in the sum of the products of perpendicular diameters of the enhancing lesion in T1 post, when compared with baseline or smallest tumor measurement (nadir). Additionally, progression may be observed by a significant increase in T2/FLAIR non-enhancing lesion on stable or increasing doses of corticosteroids compared with nadir. Here we evaluate tumor volume dynamics in T1 post MRI measurements that recently demonstrated correlation with response^12^.

A subset of 16 trial patients (both bevacizumab naive and pretreated) with tumor measurements beyond the time of progression (i.e. tumor regrowth) was used in this study. Patients excluded from this analysis either left the trial due to reasons other than tumor progression, or their tumor regrowth was not quantified (Fig. 1B). For the selected group, four to ten (median six) post treatment data points were included in this study. Table 1 summarizes the patient characteristics.

**Table 1:** Characteristics of the 16 included patients. *WT* wild type, *Surg* surgery, *TMZ* temozolomide.

Patient	Age range (years)	Initial treatment	WHO grade	IDH1	MGMT	Time to recurrence (months)
1	50–59	Surg + TMZ + trial	4	WT	Methylated	15
2	50–59	Surg + TMZ + 60 Gy	4	WT	Methylated	35
3	60–69	Surg + TMZ + 60 Gy	4	WT	Methylated	11
4	60–69	Surg + TMZ + 60 Gy	4	WT	Unmethylated	22
5	40–49	Surg + TMZ + 60 Gy	4	WT	n/a	12
6	50–59	Surg + TMZ + 60 Gy	4	WT	Methylated	5
7	50–59	Surg + TMZ + 60 Gy	4	WT	Unmethylated	10
8	50–59	Surg + TMZ + 60 Gy	4	WT	Methylated	13
9	50–59	Surg + TMZ + 59.4 Gy	4	WT	Unmethylated	16
10	50–59	Surg + TMZ + 60 Gy	4	Mutated	Methylated	21
11	50–59	Surg + TMZ + 60 Gy	4	WT	Methylated	5
12	50–59	Surg + TMZ + 72 Gy	4	WT	Methylated	7
13	50–59	Surg + TMZ + 60 Gy	4	WT	Unmethylated	6
14	50–59	Surg + TMZ + 60 Gy	4	WT	Methylated	9
15	50–59	Surg + TMZ + 60 Gy	3	Mutated	Methylated	43
16	50–59	Surg + TMZ + 60 Gy	4	WT	Methylated	31

### Mathematical model

The aim of this study was to provide a simple mathematical framework to (i) fit the observed tumor growth response data to HFSRT given in five daily fractions, and, based on this description, to (ii) simulate intermittent radiation treatment (iRT) schedules. The presented model captures only the key mechanisms of treatment response to limit the mathematical complexity of the model to be able to obtain high confidence fit parameters estimates. We extended a mathematical tumor-growth inhibition model reported by Glazar et al. to account for the contribution of HFSRT to tumor volume reduction^12^. Tumor volume growth was described as exponential growth at rate $\lambda$ [day$^{-1}$], hence neglecting potential plateauing effects due to limited carrying capacity of a tumor^28^ within this time frame. Upon treatment initiation, the effect and onset of resistance to bevacizumab and pembrolizumab treatment was modeled as previously described^12^ by exponential tumor volume reduction with rate $\gamma$ [day$^{-1}$]:

1 $$\begin{aligned} \frac{dV_l}{dt} = \lambda V_l - \gamma (t)V_l. \end{aligned}$$

Here, $V_l(t)$ is the viable tumor volume at time *t*, and $\gamma (t)$ denotes the volume decay due to bevacizumab and pembrolizumab treatment. As treatment resistance builds up, this decay rate exponentially decreases at a characteristic rate $\varepsilon$:

2 $$\begin{aligned} \frac{d\gamma }{dt} = -\varepsilon \gamma . \end{aligned}$$

In summary, this leads to the following analytic solution to this system of ODEs:

3 $$\begin{aligned} V_l(t) = V_{0,l}\cdot e^{\lambda (t-t_0) + \frac{\gamma _0}{\varepsilon }(e^{-\varepsilon (t-t_0)}-1)} \end{aligned}$$

with initial conditions $V_{0,l}$, and $\gamma _0$ at time $t_0$.

To model radiotherapy effects, at each treatment fraction delivery ($t_{RT}$), a proportion of $(1-S)$ of the viable tumor ($V_l$) is transferred to a dying compartment, $V_d$. The surviving fraction *S* is here used as a model parameter in itself, rather than as a function of radiation dose and patient-specific radiation sensitivity, as described by the linear-quadratic model^29^.

4 $$\begin{aligned} \begin{aligned} V_l(t^+_{RT})&= S\cdot V_l(t^-_{RT})\\ V_d(t^+_{RT})&= V_d(t^-_{RT}) + (1-S)V_l(t^-_{RT}). \end{aligned} \end{aligned}$$

Here, $t^-_{RT}$ denotes time immediately before delivery of a radiation fraction, $t^+_{RT}$ the time immediately after treatment delivery. By restricting ourselves to the same fraction size as the HFSRT treatment, the presented model provides a worst case estimate of no explicit consideration of radiation-induced immune stimulation.

We model radiation induced cell death as mitotic catastrophe^30^ which is a proliferation-dependent process. Hence, we describe the volume change as an exponential reduction of $V_d(t)$. Whereas others included separate parameters for the relevant tumor shrinkage rate^8,13,31^, we assume a reduction of $V_d(t)$ at rate $\lambda$ identical to the growth rate to restrict the number of free parameters. This assumption is motivated on the possibility of cell death upon the attempt of cell division^32^.

5 $$\begin{aligned} \frac{dV_d}{dt}= -\lambda V_d \quad \xrightarrow {} \quad V_d(t)=V_{0,d}e^{-\lambda t}. \end{aligned}$$

Hence, the total, observed tumor volume *V*(*t*) comprises a proliferating ($V_l(t)$) and dying ($V_d(t)$) population.

6 $$\begin{aligned} V(t) = V_l(t) + V_d(t). \end{aligned}$$

In total, this model comprises five parameters ($V_0$, $\lambda$, $\gamma _0$, $\varepsilon$, *S*; Table 2). Following previous work^12^, we evaluated the possibility of reducing the number of patient-specific parameters by considering global, patient-uniform parameters as well as functional dependencies between parameters. Using the Akaike Information Criterion (AIC)^33^ we identified the minimum AIC with a global, patient-uniform growth rate $\lambda$ and $\gamma _0=\lambda$ (Supplementary Fig. S1). Sensitivity analysis of the patient-specific parameters (*S*, $\varepsilon$, $V_0$) demonstrated the rate at which patients develop resistance to combination therapy with bevacizumab and pembrolizumab, $\varepsilon$, to be the most sensitive model parameter, and the initial tumor volume, $V_0$ to be the least sensitive (Supplementary Fig. S2).

**Table 2:** Overview of the model parameters, relevant fit bounds and range of data used for fitting (patient-specific, or all patients as a whole). We allowed for a wide range of growth rates with biologically reasonable limits^34^. Bounds for $\varepsilon$ followed previous work^12^, and the full range of possible values was used for the surviving fraction *S*. A 30% volume deviation was considered for the initial tumor volume.

Parameter	Unit	Meaning	Fit bounds	Patient-specific
$\lambda$	day$^{-1}$	Tumor growth rate in the absence of treatment	[0.017, 0.14]	–
$\varepsilon$	day$^{-1}$	Evolution of resistance rate	[0, 0.1]	$\checkmark$
*S*		Radiation surviving fraction	[0, 1]	$\checkmark$
$V_0$	cm$^3$	Pretreatment tumor volume	$V_0\times [0.7,1.3]$	$\checkmark$

### Parameter fitting and uncertainty estimation

All calculations and modeling were performed in MATLAB version 2020a. Agreement between clinically measured ($V_{measured}$) and simulated ($V_{sim}$) data was assessed by root mean squared error (RMSE) calculated over all *N* data points at times, $t_i$, including pre-treatment and four to ten (median six) post-RT measurements:

7 $$\begin{aligned} \textit{RMSE} = \sqrt{\frac{\sum _{i=1}^{N}(V_{measured}(t_i)-V_{sim}(t_i))^2}{N}}. \end{aligned}$$

Since more than one pre-treatment volume measurements were only available for a small subset of patients (7/16), a grid search was performed to identify the most suitable growth rate for the patient population as a whole. Doubling times ranging from 5 to 40 days ($\lambda =[0.017,0.14]\, {\text {day}}^{-1 }$)^34^ were used for model fitting under the constraint of a global, patient-uniform growth rate. For each growth rate, the sum of mean, median, minimum and maximum taken over all RMSEs obtained over the full data range for all patients was compared, to identify the optimal growth rate. All further results were based on this optimal growth rate.

Parameters were fitted to all data for each patient by minimizing the sum of relative squared differences between simulated and measured tumor volume. We used the MATLAB function *fmincon* with a *sqp* solver, a step tolerance of $10^{-9}$, and an optimality tolerance of $10^{-9}$. All fits converged based on these cut-off criteria.

Parameter variation due to contouring uncertainty was estimated by bootstrapping. Each data point was shifted by multiplication with a random number drawn from a normal distribution of mean one and standard deviation 0.2 (hence assuming up to 20% uncertainty) before repeating the fit of $\varepsilon$ and *S*. We assumed a maximum contouring uncertainty of 20% due to inter-observer variation, and potential microscopic infiltration of tumour cells which was not accounted for in the original GTV definition. This was a conservative estimate based on inter observer variation reported to range between 12 and 55% (mean 27%)^35^. For data points below 2 ${\text {cm}}^3$ a 0.5 ${\text {cm}}^3$ uncertainty was used, independent of the recorded tumor volume to account for a minimal contouring uncertainty. Any potential negative data points were assigned to zero volume. Fifty bootstraps were calculated, and envelopes of the obtained results are shown as uncertainty bands. For all model parameters, data are given as results of the undisturbed data with standard deviations over the bootstrap results. Correlation between model parameters, and between a patient’s gEUD and estimated surviving fraction *S* were evaluated with MATLAB’s *corrcoef* function.

### Comparison of alternative treatment schedules

We first investigate the non-inferiority of intermittent RT vs. HFSRT: The values of all parameters (*S*, $\varepsilon$, $V_0$) are assumed to take the same value for HFSRT, iRT, and iRT + boost. Based on these, the volumetric growth trajectory for each patient is simulated for five treatment fractions given intermittently every 6 weeks. The relevant patient-specific dose per fraction in terms of gEUD and D98% are listed in Suppementary Table S1. The interval of 6 weeks (42 days) corresponded to the imaging interval performed during the NCT02313272 trial and was a compromise between imaging cost and longitudinal coverage of growth observation. Previous work in low grade glioma recommended an optimal treatment interval between 30 and 60 days depending on the specific tumor growth rate^11^. Results were also analyzed for simulations of intermittent RT intervals of 4,8, and 10 weeks (28, 56, and 70 days). To account for treatment resistant tumors that may outgrow their pre-treatment size within the 6-week inter-fraction window, we also model iRT plus a three fraction boost at time of progressions (delivered in three consecutive days). Also in this scenario, the parameter values of $\varepsilon$, $V_0$, and *S* (for all fractions and boosts) are assumed to be the same as HFSRT. As neither RANO or RECIST^36^ were developed for intermittent therapy that does not aim to debulk the tumor and minimize the nadir, here we define “progression” as the tumor volume at the six-weekly assessment points exceeding the minimum measured tumor volume by more than 20%. Differences in the obtained fit parameters between patients where iRT (with or without boost) was inferior to HFSRT and those where it was equal or superior are compared by Wilcoxon rank test using MATLAB’s *ranksum* function.

Based on the assumption of a superior repair capacity of healthy relative to tumor tissue^37^, the intermittent treatment delivery holds the potential for reduced normal tissue toxicity at the same number of treatment fractions. In a second step, we hence investigate the potential gain in time to progression by increasing the number of intermittently delivered treatment fractions. We perform simulations for up to 13 treatment fractions.

The efficacy of any treatment schedule is evaluated based on time to progression by a Kaplan–Meier-analysis. Depending on the fractionation scheme the change in tumor volume varies significantly and a metric assessing both volume maintenance, and tumor eradication treatments is key. We hence score the time to reach the last recorded tumour volume (cut-off volume), assuming that this provided an estimate of the patient’s maximum tolerated tumor burden. If a patient left the trial for reasons other than increasing tumor burden, the time to reach a simulated 20$\%$ increase above the initial volume was scored. Kaplan–Meier plots were generated using the MatSurv package^38^ and log-rank p-values were calculated between HFSRT and iRT+boost treatments.

### Ethical statement

All methods were carried out in accordance with relevant guidelines and regulations. The NCT02313272 (05/12/2014) was carried out at the Moffitt Cancer Center, Tampa, FL, between August 2015 and March 2018. All patients provided written consent and the treatment protocol and any of its amendments were approved by the institutional review board of the Moffitt Cancer Center (IRB study #: Pro00014674 and # 00000971).

## Results

### Model fit to data

**Figure 2:**
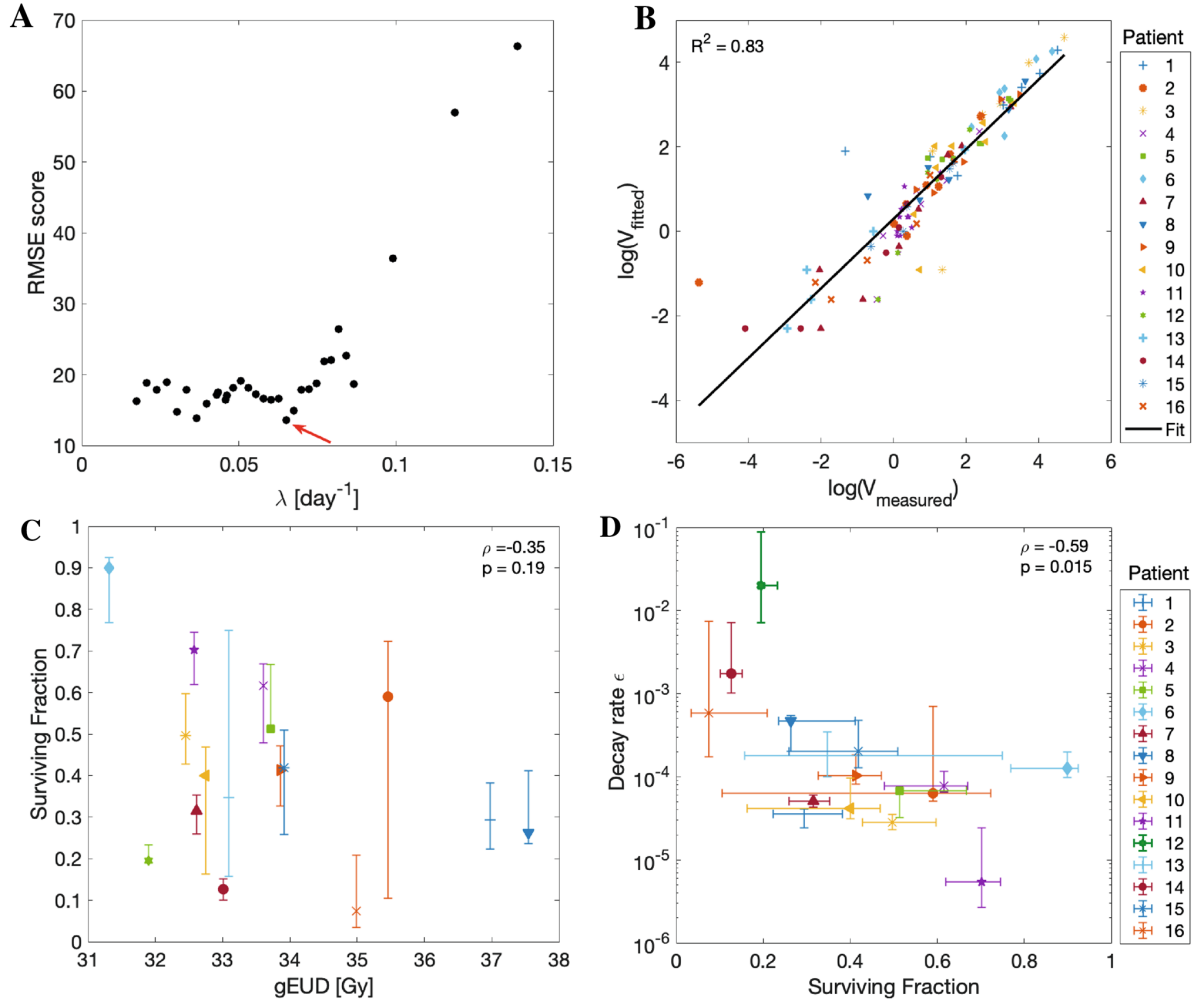
Model fit results. (**A**) Grid search results to identify the optimal growth rate $\lambda$ for the patient population (indicated by red arrow). Results of the sum over the median, mean and maximum RMSE are shown (denoted as RMSE score in cm$^3$). (**B**) Overview of the measured vs. simulated tumor volume. (**C**) Correlation analysis of the surviving fraction and the PTV gEUD. The Pearson correlation coefficient $\rho$ and corresponding p-value *p* are given. For *S* fit parameters to undisturbed data are shown with 90% confidence intervals estimated from bootstraps. See (**D**) for legend. (**D**) Correlation analysis of the logarithm of the decay rate ($log(\varepsilon )$) and the surviving fraction. Fit values to undisturbed data are shown with 90% confidence intervals over the bootstraps. *RMSE* Root mean squared error, *PTV* Planning target volume, *gEUD* generalized equivalent uniform dose.

The optimal growth rate was estimated by grid search to be $\lambda =0.065$ day$^{-1}$, corresponding to a doubling time of 11 days (Fig. 2A). For the data fits of all 16 patients with fixed $\lambda$, we obtained a median (minimum, maximum) RMSE of 1.9 (0.2, 9.7) cm$^3$. Considering the overall correlation of fitted and measured tumor volume (Fig. 2B) there was good agreement in terms of coefficients of determination ($R^2 = 0.83$) which we calculated here over the logarithms of the data points to prevent an over-representation of large values in this analysis. Model fits to individual patient data are shown in the appendix (Supplementary Fig. S4).

The obtained patient-specific fit parameters, reported as median and full range over all patients, spanned a fairly large interval reflecting the biological heterogeneity: $S = 0.41$ (0.07, 0.90), $\varepsilon = 0.9$ (0.05, 202)$\times 10^{-4}$. Interestingly, there was no correlation ($p>0.05$) between estimated surviving fraction and planned dose in terms of gEUD (Fig. 2C) or D98% (not shown) in the PTV, possibly reflecting the radiosensitivity heterogeneity of the tumors. Figure 2D shows that there was a significant negative correlation ($\rho =-0.59$, p = 0.02) between *S* and log($\varepsilon$) indicating that more radioresistant tumors, which are characterized by a high surviving fraction *S*, had a smaller rate of resistance development than non-RT treatments $\varepsilon$.

### Demonstrating non-inferiority of iRT + Boost

**Figure 3:**
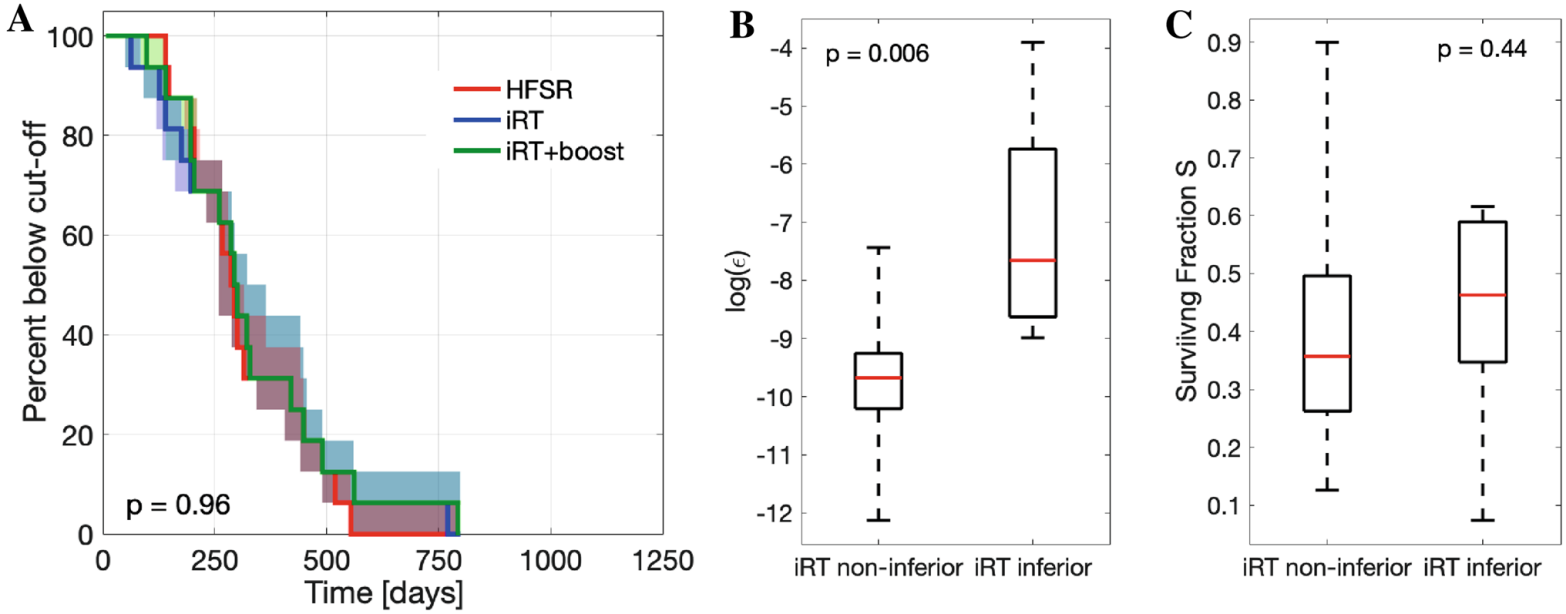
Evaluation of non-inferiority of iRT +/− boost vs HFSRT. (**A**) Kaplan–Meier plot for five treatment fractions delivered as HFSRT (red), iRT (blue) or iRT + boost (green). Shaded areas correspond to the envelope of the bootstrap estimated modeling uncertainty. The logrank test p-values is given for comparison of HFSRT and iRT + boost. (**B**) Boxplot of the logarithm of the decay rate parameter $\varepsilon$ for iRT responders and non-responders. (**C**) Boxplot of the surviving fraction *S* for iRT responders and non-responders. In (**B**) and (**C**) median values (red lines), 25th and 75th percentiles (box upper and lower bounds) and full ranges (whiskers) all calculated over patient fit values to undisturbed data are shown. Indicated p-values correspond to Wilcoxon signed-rank test.

We observed no significant difference in the Kaplan–Meier analysis between HFSRT and iRT (log-rank p-value p = 0.83), or HFSRT and iRT + boost (log-rank p-value p = 0.96) for five treatment fractions (Fig. 3A). Given the small number of patients, it is, however, also important to evaluate the individually observed differences at the patient level. In 11/16 patients, iRT was non-inferior to HFSRT, whereas time to volume cut-off was smaller for 5/16 patients. These patients (#6,8,12,14,15) were characterized by a significantly faster decay of the non-RT treatment effects (median values and full ranges: $\varepsilon _{iRT<HFSRT}$ = 4.7 (1.3, 202)$\times 10^{-4}$, $\varepsilon _{iRT\ge HFSRT}$ = 0.6 (0.1, 5.9)$\cdot 10^{-4}$, p-value between log($\varepsilon$) = 0.006) leading to faster regrowth (Fig. 3B). There was no significant difference in the obtained radiosensitivity of the two groups (median values and full ranges: $S_{iRT<HFSRT}$ = 0.26 (0.13, 0.90), $S_{iRT\ge HFSRT}$ = 0.41 (0.07, 0.70), p = 0.44) (Fig. 3C).

For four of these five patients time to progression could be prolonged by delivering a three-fraction boost once regrowth occurred. As such, Kaplan-Meier analysis (Fig. 3A) shows that for 15 out of the 16 cases, iRT + boost was non-inferior to the trial’s HFSRT treatment schedule. The single patient for whom iRT + boost was inferior to HFSRT treatment (# 12) displayed the largest resistance evolution rate $\varepsilon _{pat 12} = 201(72, 888) \times 10^{-4}$ day$^{-1}$ (fit result to undisturbed data with 90% confidence intervals over bootstraps).

### Modeling of extended intermittent treatments

**Figure 4:**
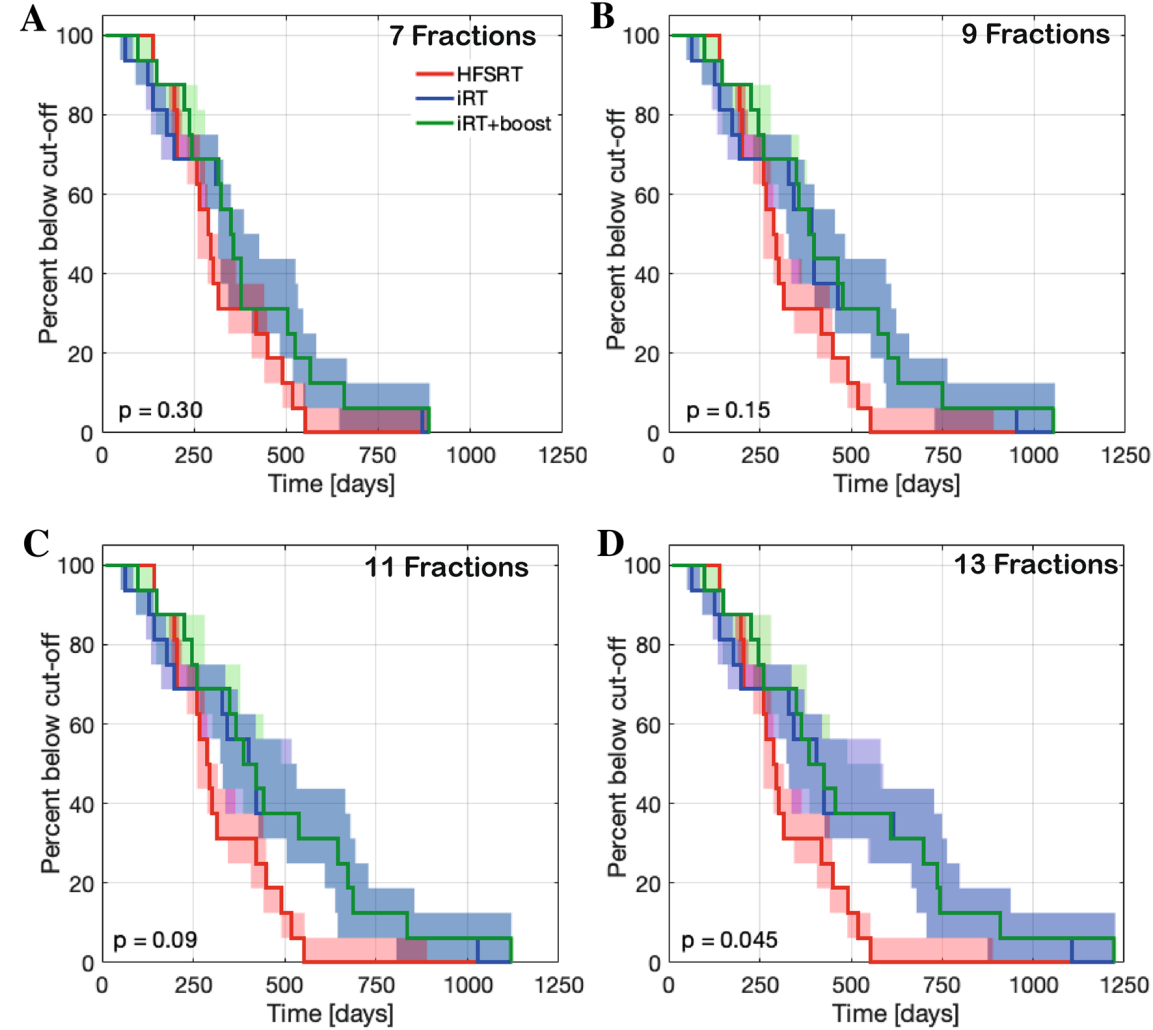
Kaplan–Meier plots for treatments with increasing maximum number of iRT fractions. Shown are fitted HFSRT (red), and simulated iRT (blue) and iRT + boost (green) results. Shaded areas correspond to the envelope of the bootstrap estimated modeling uncertainty. The logrank test p-values are given for comparison of HFSRT and iRT+boost. (**A**) Up to seven fractions. (**B**) Up to nine fractions. (**C**) Up to eleven fractions. (**D**) Up to thirteen fractions.

Normal and tumor tissue radiosensitivity may vary strongly between individual patients. The opportunity to either continue or interrupt treatment at each of the 6-weekly evaluation time-points holds great potential for treatment personalization. The total number of delivered fractions can be adjusted to enable personalized dose escalation given the absence of acute normal tissue toxicity. Although we do not score normal tissue complication in this model, it is suggested that in the intermittent setting normal tissue may be capable to compensate for radiation-induced damage more effectively than the tumor^37,39,40^. This motivates an escalation of the total delivered dose in the iRT setting. We hence simulated alternative treatments allowing up to 13 treatment fractions as a hypothesis generating study. As the number of fractions increases, the time to reach the cut-off volume is continuously prolonged provided the subject’s tumor shows no regrowth, which in turn increases separation of the Kaplan–Meier curves as shown for up to seven (Fig. 4A), nine (Fig. 4B), 11 (Fig. 4C) , or 13 (Fig. 4D) treatment fractions. However, given the small cohort size, differences between HFSRT and iRT + boost for up to eleven intermittent treatment fractions were not significant as assessed by logrank testing ($p\ge 0.05$). Only a subset of five patients (#1, 3, 7, 10, 11) would benefit from $>11$ treatment fractions (Fig. 4D) leading to a further separation of the Kaplan–Meier plots for observation time above 550 days, and significantly better iRT + boost treatment compared to HFSRT ($p=0.045$). Changing the timing between intermittent radiation fractions did not demonstrate a significant difference in results. However, prolonging inter-fraction times beyond 6 weeks may diminish potential benefits of iRT as the tumor regrowth could lead to progression before subsequent radiation (Supplementary Fig. S3).

**Figure 5:**
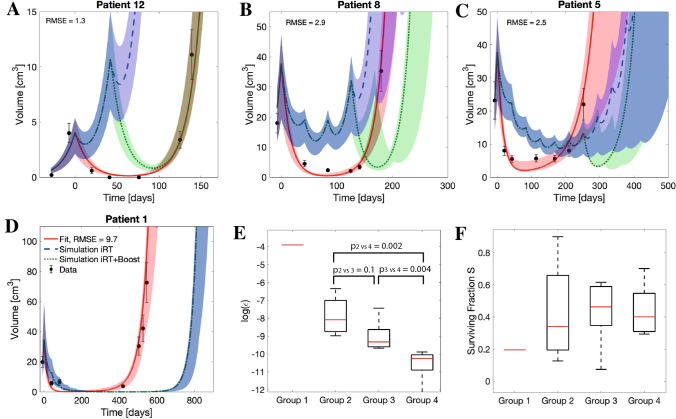
Grouping of patient response. (**A**–**D**) Estimated growth trajectories of representative patients for fitted HFSRT (red), and simulated iRT (blue) and iRT + boost (green) treatments with up to eleven treatment fractions. Shaded areas correspond to the envelope of the bootstrap estimated modeling uncertainty. (**A**) Group 1, (**B**) Group 2, (**C**) Group 3, (**D**) Group 4. (**E**) Analysis of the logarithm of the decay rate $\varepsilon$ for the different groups. Indicated p-values correspond to Wilcoxon signed rank tests between the groups comprising more than one patient. (**F**) Analysis of the radiotherapy surviving fraction for the different groups. There were no significant differences. In (**E**) and (**F**) median values (red lines), 25th and 75th percentiles (box upper and lower bounds) and full ranges (whiskers) are shown.

By comparing the individual patient’s response between protocols (see Tables 3 and 4) we identified four subgroups of patients with respect to their response to the different protocols: Group (1) HFSRT is best (# 12), Group (2) iRT is inferior to HFSRT, but iRT + Boost compensates this difference (# 6, 8, 14, 15), Group (3) iRT + boost further prolongs time to progression (# 2, 4, 5, 9, 13), Group (4) iRT is best (# 1, 3, 7, 10, 11, 16). Figure 5A–D shows individual growth trajectories for representative examples for each of these groups for a scenario of up to eleven iRT fractions to clearly visualize the differences in growth response. The full set of growth trajectories is given in the supplementary material (Supplementary Fig. S4). The relevant evaluation of the model parameters for these sub-groups is shown in Fig. 5E,F. While there was no significant difference between the radiotherapy surviving fractions, the groups differed in their fit results for parameter $\varepsilon$ with small decay rates corresponding to a benefit from iRT treatment. The logarithm of epsilon differed significantly between groups 2 and 4 ($p_{2 vs. 4} = 0.006$), and 3 and 4 ($p_{3 vs. 4} = 0.02$), respectively.

**Table 3:** Overview of the predicted times to progression ($\tau$) in days for HFSRT and iRT. Values obtained for fits to undisturbed data are shown together with bootstrap uncertainty ranges for the indicated numbers of fractions delivered intermittently every 6 weeks.

Patient	#Fractions					
	$\tau _{HFSRT}$ (days)	$\tau _{iRT}$ (days)				
	5	5	7	9	11	13
1	546 (546, 546)	546 (546, 546)	641 (637, 655)	727 (719, 753)	806 (794, 842)	879 (865, 926)
2	253 (251, 268)	253 (253, 268)	300 (253, 339)	320 (253, 412)	320 (253, 511)	320 (253, 552)
3	482 (482, 482)	482 (482, 482)	555 (537, 569)	622 (584, 648)	682 (644, 721)	739 (672, 790)
4	276 (276, 276)	276 (276, 276)	312 (307, 322)	327 (321, 362)	327 (320, 362)	327 (320, 362)
5	274 (252, 352)	274 (252, 352)	327 (303, 416)	373 (324, 472)	373 (324, 506)	373 (324, 570)
6	133 (133, 133)	123 (107, 129)	123 (107, 129)	123 (107, 129)	123 (107, 129)	123 (107, 129)
7	434 (427, 446)	434 (427, 446)	515 (508, 534)	588 (579, 612)	655 (642, 684)	718 (701, 751)
8	182 (179, 204)	156 (153, 202)	156 (153, 202)	156 (153, 201)	156 (153, 202)	156 (153, 202)
9	263 (222, 284)	263 (222, 284)	317 (246, 339)	363 (249, 388)	363 (249, 409)	363 (246, 409)
10	407 (399, 439)	407 (399, 439)	486 (477, 524)	556 (542, 598)	620 (601, 668)	679 (655, 738)
11	676 (332, 875)	676 (332, 875)	796 (393, 1027)	901 (446, 1160)	995 (495, 1280)	1082 (538, 1391)
12	139 (139, 139)	38 (36, 75)	38 (35, 75)	38 (35, 75)	38 (35, 75)	38 (35 , 75)
13	308 (308, 308)	308 (308, 308)	351 (308, 376)	392 (308, 444)	411 (308, 510)	411 (320, 577)
14	188 (188, 200)	123 (79 , 162)	123 (79 , 162)	123 (79 , 162)	123 (79 , 166)	123 (79 , 161)
15	195 (170, 256)	192 (122, 215)	192 (123, 304)	192 (122, 236)	192 (122, 325)	192 (122, 325)
16	271 (248, 429)	271 (248, 429)	353 (330, 512)	433 (396, 586)	514 (457, 653)	594 (418, 716)

**Table 4:** Overview of the predicted times to progression ($\tau$) in days for HFSRT and iRT + boost. Values obtained for fits to undisturbed data are shown together with bootstrap uncertainty ranges for the indicated numbers of fractions delivered intermittently every 6 weeks.

Patient	#Fractions					
	$\tau _{HFSRT}$ (days)	$\tau _{iRT+boost}$ (days)				
	5	5	7	9	11	13
1	546 (546, 546)	546 (546, 546)	641 (637, 655)	727 (719, 753)	806 (794, 842)	879 (865, 926)
2	253 (251, 268)	253 (253, 268)	300 (253, 339)	343 (253, 4167)	363 (253, 511)	363 (253, 416)
3	482 (482, 482)	482 (482, 482)	555 (537, 569)	622 (584, 648)	682 (644, 721)	739 (672, 790)
4	276 (276, 276)	276 (276, 276)	312 (307, 322)	344 (322, 364)	344 (322, 364)	344 (322, 364)
5	274 (252, 352)	274 (252, 352)	327 (303, 416)	374 (347, 472)	396 (372, 506)	396 (372, 570)
6	133 (133, 133)	133 (132, 133)	138 (135, 143)	138 (135, 143)	138 (135, 143)	138 (135, 143)
7	434 (427, 446)	434 (427, 446)	515 (508, 534)	588 (579, 612)	655 (642, 684)	718 (701, 751)
8	182 (179, 204)	181 (179, 202)	227 (219, 247)	227 (219, 267)	227 (219, 267)	227 (222, 267)
9	263 (222, 284)	263 (222, 284)	317 (270, 339)	365 (295, 388)	388 (295, 432)	388 (292, 432)
10	407 (399, 439)	407 (399, 439)	486 (477, 524)	556 (542, 598)	620 (601, 668)	679 (655, 738)
11	676 (332, 875)	676 (332, 875)	796 (393, 1027)	901 (446, 1160)	995 (495, 1280)	1082 (540, 1391)
12	139 (139, 139)	38 (36, 139)	38 (35, 139)	38 (35, 139)	38 (35, 139)	38 (35, 139)
13	308 (308, 308)	308 (308, 308)	351 (308, 376)	372 (308, 444)	372 (308, 477)	372 (312, 477)
14	188 (188, 200)	188 (79, 195)	220 (79, 257)	220 (79, 257)	220 (79, 267)	220 (79 , 253)
15	195 (170, 256)	195 (123, 215)	237 (123, 304)	237 (123, 280)	237 (123, 369)	237 (123, 369)
16	271 (248, 429)	271 (248, 429)	353 (330, 512)	433 (396, 586)	514 (429, 653)	594 (419, 716)

## Discussion

Improving treatment responses and outcomes of rHGG patients is an unmet clinical need. Here we developed, analyzed, and calibrated a mathematical model to simulate and compare HFSRT with 6 Gy $\times$ 5 fractions delivered in one week to intermittent radiation of 6 Gy in 5 or more fractions delivered intermittently at 6 week intervals with concurrent bevacizumab and pembrolizumab. Model simulation results suggest that the intermittent treatment plus boost should be equal to or superior to the five daily fraction HFSRT protocol for all but one of the evaluated 16 patients. We found that the parameter characterizing the rate of developing resistance to bevacizumab and pembrolizumab treatment was an indicator of the efficacy of iRT. Patients whose tumor developed resistance slowly would benefit most from iRT compared to HFSRT. Based on our data it was not possible to identify these patients from pre-treatment or early evaluation time points, however, it is conceivable that other biomarkers, e.g. genetic data or imaging radiomics, could be predictive of this mechanism and hence allow for stratification of patient according to the best suitable fractionation scheme. In the absence of such predictive markers, it would hence be essential to continuously monitor the tumor’s response as suggested here. We showed that iRT could possibly extend the time to tumor progression by allowing for personalization of later treatment fractions based on the observed response to the previous fraction. This could be done, as suggested here, through the option of a boost in case of progression, or by adapting the dose per fraction regimen at a personalized level as previously suggested for other tumor locations^23,28^. Fraction size variation was not addressed in this model due to the limited amount of data available. Given a more in-depth knowledge of the specific radiosensitivity parameters per tumor, for example based on genetic information, further analyses could integrate a tumor-specific radiosensitivity index^41,42^. We intended to restrict this analysis to a purely data-driven estimation of response with few patient-specific fit parameters. Genetic or molecular data was not available and hence could not be accounted for. Despite these simplifications, an interesting observation was that more radioresistant tumors generally displayed a smaller rate of resistance development than non-RT treatments leading to a significant correlation of *S* and $\varepsilon$. Since RT and immunotherapy were delivered simultaneously in the NCT02313272 trial, it cannot be determined if this is a biological result or due to mathematical parameter non-identifiability. It is generally agreed that the extent of the RT-induced immune stimulus is dose dependent, and hence proportional to the number of cells killed^14^. It could be speculated that a smaller immune stimulus resulted in a delay of the onset of treatment resistance however further biological, clinical, and mathematical analyses are needed in order to draw definite conclusions.

The proposed mathematical model, despite using only three patient-specific parameters, provided an acceptable fit to the data. Akaike Information Criteria analysis showed that allowing for all five parameters to be patient-specific may lead to better fits to the clinical data, but at the cost of overfitting of the interdependent model parameters given the sparse patient data (median six on-treatment data points per patient). Adding additional biological complexity, such as radiation-induced immune stimulation, albeit of high clinical relevance, would even further limit the identifiability of parameters. By choosing a deliberately simple mathematical description, the number of fitted patient-specific parameters and modeling uncertainty was optimally balanced for the small clinical cohort of 16 patients. This also limited the power of our analysis leading to no statistical significance of a potential benefit of iRT vs. HFSRT for less than 13 treatment fractions. Given the observed separation of the Kaplan-Meier plots for seven to 13 treatment fractions, however, the herein presented results motivate further evaluation on a larger set of patients. There are a number of potential benefits of iRT over HFSRT which we will discuss in the following together with the relevant assumptions made upon modeling: (i) possibility for dose escalation, (ii) treatment personalization in reaction to the observed response, (iii) repeated immune stimulation and antigen sampling, (iv) maintenance of radiosensitive tumor subpopulation, (v) tumor management rather than tumor eradication in case of a purely palliative treatment.

The clinical trial providing the data for this analysis demonstrated that it was safe to deliver 6 Gy $\times$ 5 as HFSRT with respect to normal tissue tolerance in rHGG patients^17^. Addition of bevacizumab may have played a role in decreasing incidence of cerebral edema and radiation necrosis. Normal tissue toxicity following iRT + boost would need to be investigated for treatments comprising more than five fractions. Estimates on normal tissue complication could be related to previous results from trials investigating hypofractionated radiotherapy or stereotactic radiosurgery in combination with immunotherapy for the treatment of melanoma brain metastases^43,44^. Severe radiation-induced late side effects of brain tissue may be beyond the expected life span of rHGG patients, however, acute radiation-induced side effects such as headache, seizures, intracranial haemorrhage, and brain edema^43^ should be considered. Besides these manageable toxicities, radiation necrosis may be a dose limiting factor for iRT treatments with incidence times in the order of months to few years following RT^44,45^. Acute radiation-induced toxicity may strongly correlate with the irradiated volume and dosing which together with potential normal tissue recovery between fractions makes estimations difficult. A clear advantage of the intermittent treatment approach is the option to halt further irradiation if severe acute radiation-induced toxicity occurs, which is in line with a personalized treatment approach.

Another advantage of iRT is the possibility to adapt the irradiated volume according to the observed growth. This includes local PTV adaptations, and potential inclusion of progression sites appearing outside the primary tumor location. This type of treatment paradigm would increase treatment cost due to repeated imaging and treatment planning. Recent advances in automated treatment planning^46,47^ and the delivery of the treatment under MRI guidance with an MR-Linac^48–50^ could pose a potential solution to mitigate this limitation of intermittent treatments. Response monitoring and treatment planning steps could be combined in this scenario^51^.

Additionally, intermittent RT may hold potential for synergistic action with immunotherapy due to repeated antigen re-sampling as suggested by recent (pre-) clinical studies^14–16,52,53^. Therefore, iRT is particularly promising in combination with immune-checkpoint inhibition therapy. In this approach bevacizumab and pembrolizumab treatments were modeled as additive effects to RT only. Whereas others explicitly modeled the drug administration schedule^54–56^ we used a simplified model of bevacizumab and pembrolizumab administration, ignoring (patient-) specific pharmacodynamics as previously suggested^12^ to limit the complexity of our model. Since in the NCT02313272 trial RT was only delivered in combination with bevacizumab and pembrolizumab it was not possible to separate a potential radiation-induced immune stimulation from direct radiation cytotoxicity. Since no further immune stimulation was modeled in the intermittent approach, it is suggested that the volume estimates made by our model may overestimate tumor growth dynamics and provide a worst-case scenario. While it is mathematically straightforward to describe immunostimulatory effects of radiation, the calibration and validation of the associated parameters proves infeasible and limits model predictive power^57^.

It should also be stressed that the time between radiation fractionations for iRT could allow for regrowth of both resistant and sensitive populations. Pre-clinical data and evolutionary convention^58,59^ suggest that resistant cells may display a fitness disadvantage relative to sensitive clones in the absence of the selective pressure, allowing for the sensitive subpopulation to preferentially repopulate the tumor. Treatment prolongation by delivery of additional fractions would increase tumor radioresistance. However, it is expected that intermittent treatments would provide an advantage over daily HFSRT as sensitive subclones may repopulate more effectively between fractions. This advantage would be more pronounced with increasing time between RT fractions. Pérez-García et al. specifically evaluated the impact of the treatment interval and concluded that the optimal timing depends on the individual tumor growth rate^9,11^. In our study, a constant growth rate was used for all patients due to insufficient data to estimate tumor growth in the absence of treatment at an individual level. If the pre-treatment tumor growth rate was known, personalized adaptations of the fractionation interval would be possible allowing for longer intervals in case of slow growing tumors. It should also be noted that RT-induced repeated antigen sampling may provide a specific immune stimulus targeting radio-resistant tumor subpopulations. This would be a further motivation for the combination of iRT with immune checkpoint inhibitors.

Finally, by maintaining rather than eradicating the tumor, growth is likely to be slower for larger tumours than those close to eradication as presented in the NCT02313272 trial. As such, modeling exponential tumor growth, one of the key assumptions within our model, for both the HFSRT and iRT scenarios would in the worst case overestimate the growth of larger tumors present following iRT. In summary, the assumptions in this analysis lead to a worst case estimate of the simulated treatment response following iRT, which strengthens the results presented in this hypothesis-generating study.

Patients with rHGG currently have no curative treatment options^60,61^, and radical treatment attempting tumor control may be suboptimal considering its purely palliative and life-prolonging intent. The intermittent treatment approach embraces the aim of tumor control and volume management rather than tumor eradication. This comes at the cost of potentially not improving the tumor burden, which may affect the quality of life of the patients. For this reason, iRT should be restricted to those patients with asymptomatic recurrence or those who are neurologically stable. Our hypothesis generating modelling study provides a numerical estimate of the potential gain in time to progression for a large subgroup of patients. As such, we have demonstrated the mathematical feasibility of iRT treatments for rHGG patients. This approach should be carried forward to be evaluated in a prospective clinical trial.

## Conclusions

There is a critical unmet clinical need to improve response rates and overall outcome for patients with rHGG. Based on a deliberately simple, worst-case estimate mathematical model we propose that intermittent radiotherapy treatments with an optional three fraction boost may be a safe for this group of patients with the potential for delayed time to progression. This novel radiation treatment schedule has additional potential for personalized treatment decisions in terms of geometric dose delivery and fraction size optimization based on the observed tumor response to previous fractions.

## Supplementary Information

Supplementary Information.

## Data Availability

The data sets used and/or analyzed in the study are available from the corresponding author on reasonable request. The code will be published on https://github.com/sbrueningk/iRT.git.
