# Author Correction: Intermittent radiotherapy as alternative treatment for recurrent high grade glioma: a modeling study based on longitudinal tumor measurements

**DOI:** 10.1038/s41598-022-08620-3

**Published:** 2022-03-15

**Authors:** Sarah C. Brüningk, Jeffrey Peacock, Christopher J. Whelan, Renee Brady‑Nicholls, Hsiang-Hsuan M. Yu, Solmaz Sahebjam, Heiko Enderling

**Affiliations:** 1 Department of Biosystems Science and Engineering, ETH Zürich, Basel, Switzerland; 2 Swiss Institute for Bioinformatics (SIB), Lausanne, Switzerland; 3 Department of Radiation Oncology, H. Lee Moffitt Cancer Center, Tampa, FL USA; 4 Department of Cancer Physiology, H. Lee Moffitt Cancer Center, Tampa, FL USA; 5 Department of Integrated Mathematical Oncology, H. Lee Moffitt Cancer Center, Tampa, FL USA; 6 Department of Neuro-Oncology, H. Lee Moffitt Cancer Center, Tampa, FL USA; 7 Department of Oncologic Sciences, University of South Florida, Tampa, FL USA

Correction to: *Scientific Reports* 10.1038/s41598-021-99507-2, published online 12 October 2021

The original version of this Article contained an error in the Acknowledgments section.

“The work of this project was initiated at the 2019 Integrated Mathematical Oncology workshop at Moffitt Cancer Center for which the participation of SCB was kindly sponsored by the Moffitt Physical Sciences Oncology Center (NIH/NCI U54CA143970) and the Integrated Mathematical Oncology Department. Research reported in this publication was supported by the National Cancer Institute of the National Institutes of Health under Award Number R21CA234787 (HE, SS, HY). The work of SCB was supported by the Swiss National Science Foundation Spark Grant 190647.”

now reads:

“The work of this project was initiated at the 2019 Integrated Mathematical Oncology workshop at Moffitt Cancer Center for which the participation of SCB was kindly sponsored by the Moffitt Physical Sciences Oncology Center (NIH/NCI U54CA143970) and the Integrated Mathematical Oncology Department. Research reported in this publication was supported by the National Cancer Institute of the National Institutes of Health under Award Number R21CA263911 (HE, SS, HY). The work of SCB was supported by the Swiss National Science Foundation Spark Grant 190647.”

The original Article has been corrected.

