# Supplementary material for: Intermittent radiotherapy as alternative treatment for recurrent high grade glioma: a modeling study based on longitudinal tumor measurements
Source: Sci Rep. 2021 Oct 12;11:20219. doi: 10.1038/s41598-021-99507-2 (PMC8511136; doi:10.1038/s41598-021-99507-2)
Supplement: Supplementary file 1 — Supplementary Information. [file 41598_2021_99507_MOESM1_ESM.pdf]

Intermittent radiotherapy as alternative treatment for recurrent high grade glioma: A modeling study based on longitudinal tumor measurements

Sarah C. Brüningk[1,2,*], Jeffrey Peacock[3], Christopher J. Whelan[4], Renee Brady-Nicholls[5], Hsiang-Hsuan M. Yu[3,6], Solmaz Sahebjam[7,6], and Heiko Enderling[3,5,6]

[1]Department of Biosystems Science and Engineering, ETH Zürich, Basel, Switzerland
[*]sarah.brueningk@bsse.ethz.ch
[2]Swiss Institute for Bioinformatics (SIB), Lausanne, Switzerland
[3]Department of Radiation Oncology, H. Lee Moffitt Cancer Center, Tampa, FL, USA
[4]Department of Cancer Physiology, H. Lee Moffitt Cancer Center, Tampa, FL, USA
[5]Department of Integrated Mathematical Oncology, H. Lee Moffitt Cancer Center, Tampa, FL, USA
[6]Department of Oncologic Sciences, University of South Florida, Tampa, FL, USA
[7]Department of Neuro-Oncology, H. Lee Moffitt Cancer Center, Tampa, FL, USA

Supplementary Material

Akaike Information Criterion Analysis

The Akaike Information Criterion (AIC) was calculated for each of the 16 included patients for each of the following models: Model 1) Fit V_0, S, ε, and λ per patient and $\gamma_0 = \lambda$; Model 2) Fit V_0, S, ε, γ_0 and λ per patient; Model 3) λ constant for all patients, $\gamma_0 = \lambda$, fit V_0, S, ε per patient; Model 4) Fit γ_0, V_0, S, and ε) per patient, λ constant for all patients. Here, we followed AIC calculation as described by Portet et al. based on the residual sum of squares (RSS)[1]:

$$AIC = \begin{cases} n_{params} < \frac{n_{obs}}{40} & n_{obs} \cdot log(\frac{RSS}{n_{obs}}) + 2n_{params} \\ \text{else} & n_{obs} \cdot log(\frac{RSS}{n_{obs}}) + 2n_{params} + \frac{2n_{params} \cdot n_{obs}}{(n_{obs} - n_{params} - 1)} \end{cases} \tag{1}$$

Here, the number of observations, n_{obs} refers to the number of volume measurements, n_{params} is the number of parameters, and RSS refers to the residual sum of squares between the measured ($V_{measured}$) and simulated (V_{sim}) volumes:

$$RSS = \sum_{i=1}^{n_{obs}} ((V_{measured} - V_{sim}).^2) \tag{2}$$

A subset of patients did not comprise sufficient data points to evaluate all suggested models. Patients 6, 8 and 14 had six data points, patient 15 five which impeded the calculation of the AIC for model 2 and 1,2 and 4 respectively for these patients (the relevant bars are hence missing in Figure S1). The patient contributions were averaged (accounting for the number of contributing patients) to arrive at the total AIC values: $meanAIC_{Model1} = 44.4$, $meanAIC_{Model2} = 68.7$, $meanAIC_{Model3} = 25.1$, $meanAIC_{Model4} = 40.5$
We concluded that model 3 was most suitable in terms of AIC and the number of patients which could be included.

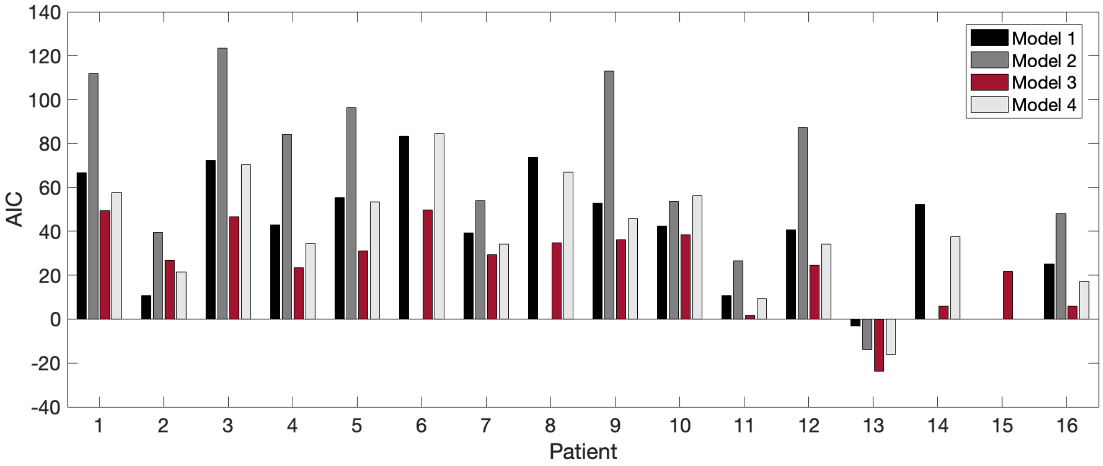

Figure S1. The Akaike Information Criterion (AIC) was calculated as described by Portet et al. based on the residual sum of squares[1] for each of the following models: Model 1) Fit V_0, S, ε, and λ per patient and $\gamma_0 = \lambda$; Model 2) Fit V_0, S, ε, γ_0 and λ per patient; Model 3) λ constant for all patients, $\gamma_0 = \lambda$, fit V_0, S, ε per patient; Model 4) Fit γ_0, V_0, S, and ε) per patient, λ constant for all patients. The obtained AIC values are shown where possible given the number of data points for each patient. Model 3 (red) provided the minimal total AIC summed over all 16 patients.

Local Sensitivity Analysis

Following previous work[2], a local sensitivity analysis of the model parameters was performed[3] to determine how the simulated tumor volume V changed in response to small perturbations in the model parameters S, ε and V_0. Mathematically, this is given by $\partial V/\partial p$, where $p = \{S, \varepsilon, V_0\}$. Specifically, using finite differences, we calculate the forward difference dense Jacobian $\partial V/\partial p$ for each patient at each measurement point in time. Depending on the evaluation time point, different model parameters are most sensitive. Four examples of the variation of local sensitivity over the evaluation time point are given in Figure S2 A). Here we observe that, as expected, pre-treatment data points are only influenced by V_0. Moreover, the sensitivity of ε increases exponentially with time since RT onset. To summarize this, we report in Figure S2 B) the normalized maximum of the sensitive scores for each variable in ranked order. Here, it is observed that ε is the most sensitive parameter, followed by the surviving fraction S, and the pre-treatment volume V_0 for all patients but #12.

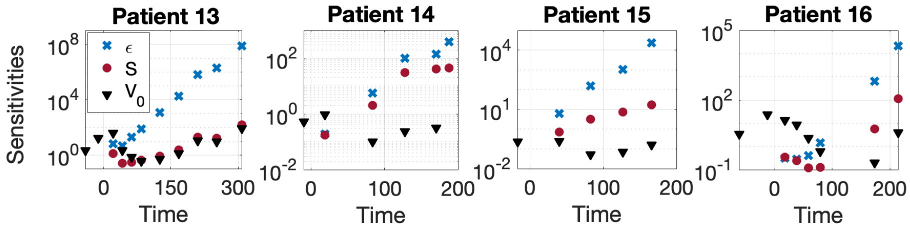

(A) Variation of local sensitivity by data point for a subset of patients.

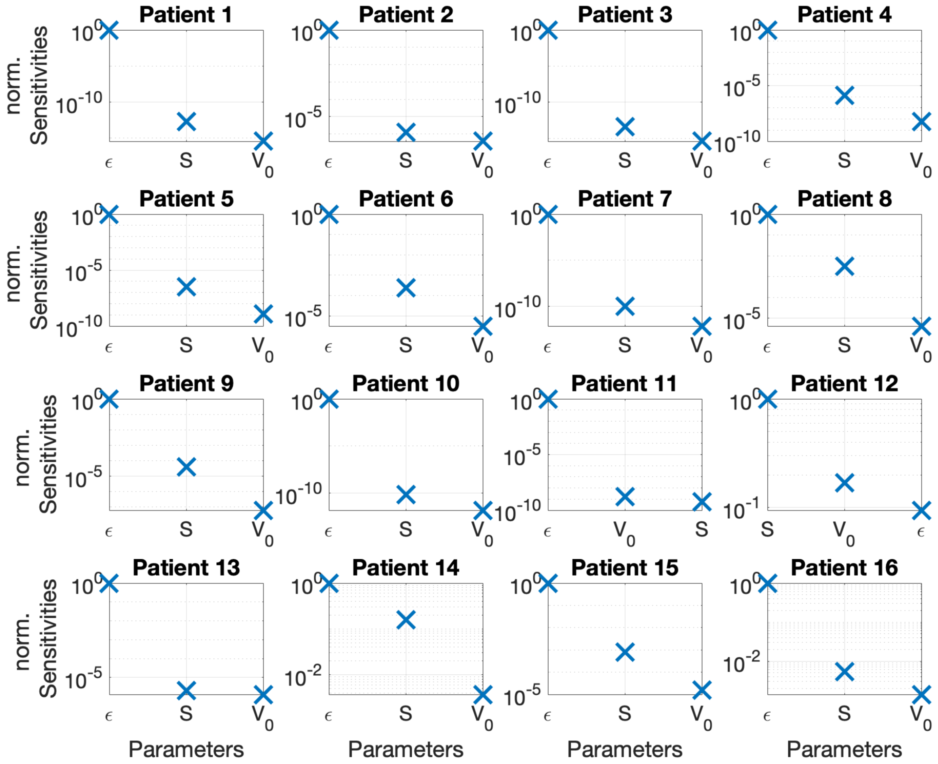

(B) Ranked, normalized sensitivity for each variable.

Figure S2. Local sensitivity analysis of the model parameters S, V_0, and ε. A) Overview over the variation of the sensitivity by data point, B) Ranked, normalized, maximum over time local sensitivity scores calculated as the partial derivatives of the tumor volume V with respect to the model parameters.

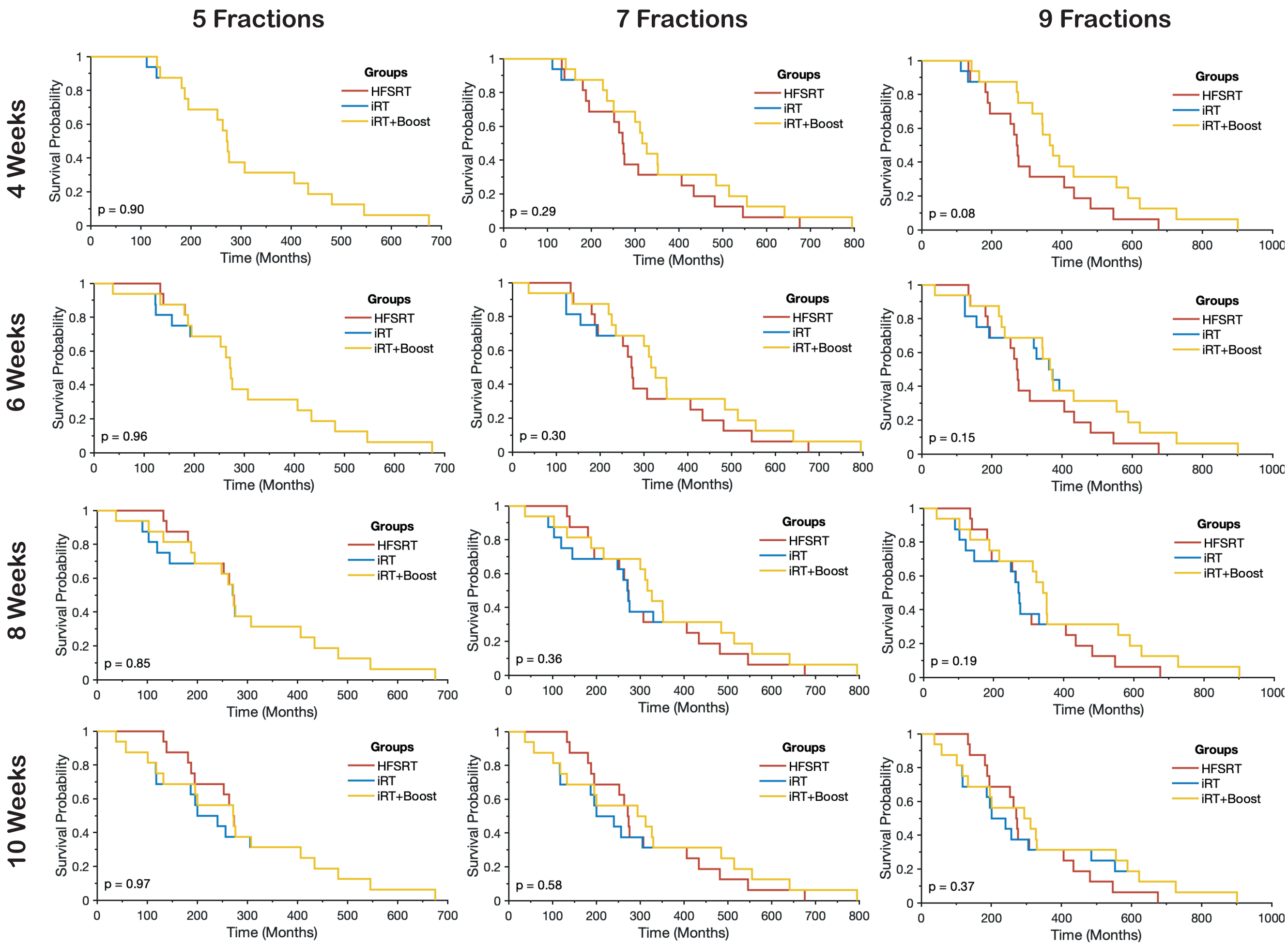

Figure S3. Overview of model predictions for different times between fractions ranging from four to ten weeks. Reported p-values correspond to log-rank testing between HFSRT and iRT+boost treatments.

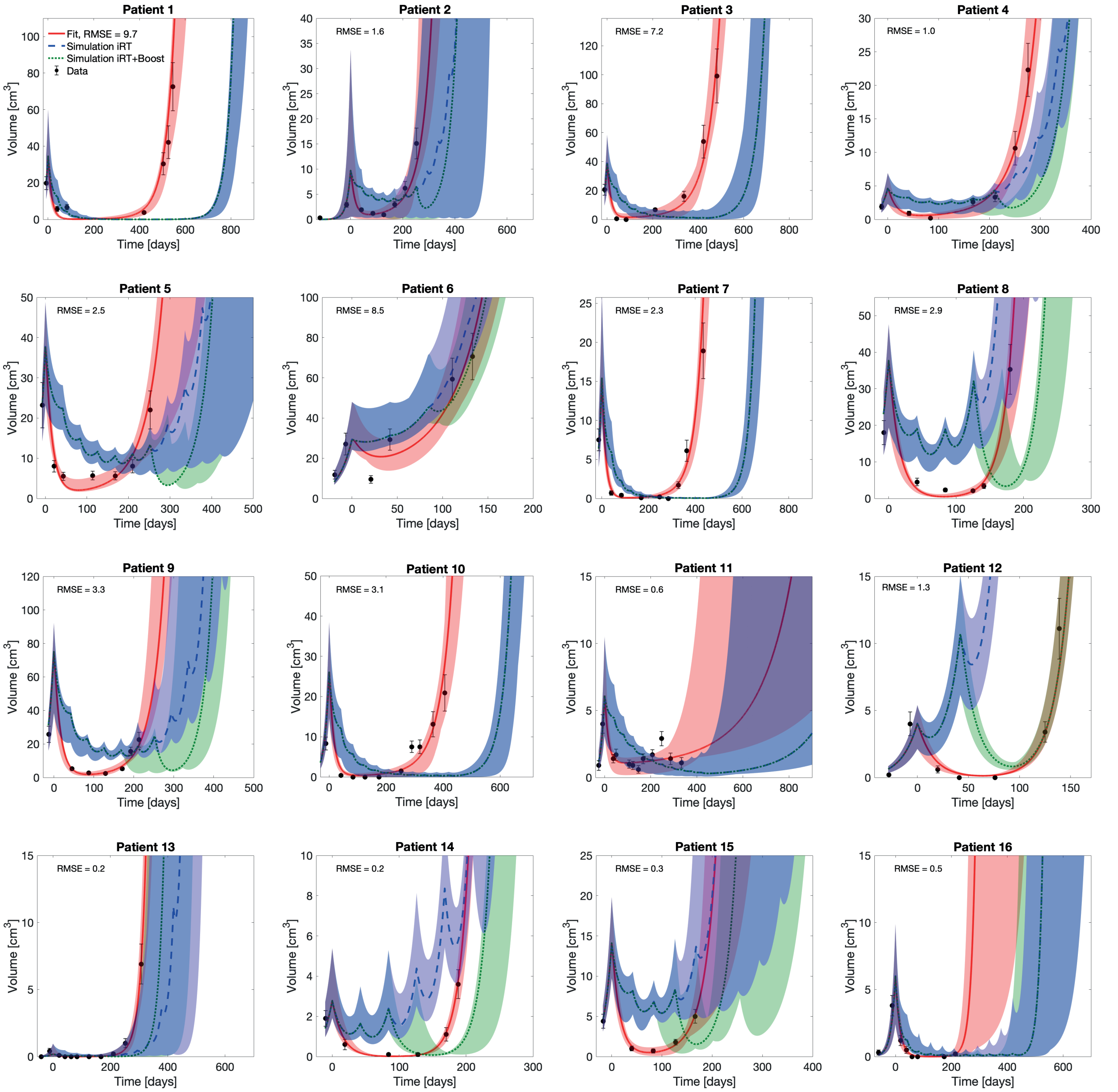

Figure S4. Estimated growth trajectories of all included patients for fitted HFSRT (red), and simulated iRT (blue) and iRT+boost (green) treatments with up to 11 treatment fractions. Shaded areas correspond to the envelope of the bootstrap estimated modeling uncertainty.

Table S1. Overview of the patient-specific dosing in terms of total PTV gEUD and D98%. A Lyman parameter of -10 was used for gEUD calculation. Treatments were delivered within five daily fractions for all patients. Abbreviations: PTV: Planning target volume, gEUD: generalized equivalent uniform dose, D98% near minimum dose at least received by 98% of the PTV.

Patient	PTV gEUD [Gy]	PTV D98% [Gy]
1	37.0	35.9
2	35.5	34.5
3	32.5	29.3
4	33.6	30.7
5	33.7	30.6
6	31.3	29.5
7	32.6	28.8
8	37.5	34.0
9	33.9	30.5
10	32.7	31.2
11	32.6	31.0
12	31.9	30.3
13	33.1	31.1
14	33.0	30.1
15	33.9	31.9
16	35.0	31.5

References

1. Portet, S. A primer on model selection using the akaike information criterion. *Infect. Dis. Model.* **5**, 111–128, DOI: https://doi.org/10.1016/j.idm.2019.12.010 (2020).

2. Brady-Nicholls, R. *et al.* Prostate-specific antigen dynamics predict individual responses to intermittent androgen deprivation. *Nat. Commun.* **11**, 1750, DOI: 10.1038/s41467-020-15424-4 (2020).

3. Banks, H. & Tran, H. T. *Mathematical and Experimental Modeling of Physical and Biological Processes* (Chapman and Hall, New York, 2009).
